# The complete chloroplast genome of agarwood producing species, *Aquilaria sinensis* (Lour.) Gilg: a species on IUCN red list

**DOI:** 10.1080/23802359.2019.1664954

**Published:** 2019-09-13

**Authors:** Ching-Ping Lin, Yuan-Ting Hsiao, Yu-Jen Andy Hsiao, Shu-Jen Chou, Ai-Ping Chen, Ching-I Kuo, Long-Fang Oliver Chen

**Affiliations:** a Institute of Plant and Microbial Biology, Academia Sinica, Taipei, Taiwan;; b Department of Earth and Life Sciences, University of Taipei, Taipei, Taiwan;; c Agarwood Research Institute, Asia (Macau) Humanities and Nature Research society, Macau, P. R. China;; d P&I Enterprise Co., Ltd., Guishan, Taiwan

**Keywords:** *Aquilaria sinensis*, Thymelaeaceae, agarwood, chloroplast genome, conservation

## Abstract

The entire chloroplast genome of *Aquilaria sinensis* (Lour.) Gilg was identified as a circular molecule of 174,885 bp length with a typical tetrad structure, including a pair of inverted repeats (42,103 bp each), a large single copy (87,331 bp) and a small single copy (3,348 bp) regions. The *A*. *sinensis* cp genome encoded 8 rRNAs, 39 tRNAs, and 90 proteins. A phylogenetic tree was reconstructed using the 43 protein-coding genes of eight Thymelaeaceae. Two other Malvales, *Abelmoschus esculentus* and *Durio zibethinus*, were selected as the outgroup. Our phylogenetic analysis suggests that the five examined species of *Aquilaria* appeared a monophyletic group with robust support.

## Main text

*Aquilaria sinensis* (Lour.) Gilg belongs to the order Malvales, family Thymelaeaceae. The species has been heavily exploited for its fragrant resin-filled heartwood known as agarwood, which has resulted in declines of at least 30% in the past 10 years. It is assumed that the population was either stable or declining prior to the last 10 years, leading to at least a 30% decline over the last three generations of this species (Harvey-Brown 2018). IUCN (International Union for Conservation of Nature and Natural Resources) therefore assessed this species as vulnerable. Genome resource banking of *Aquilaria* species would provide useful information for conservation actions such as to establish species in harvest and trade management. Hence, we complete an entire chloroplast (cp) genome sequence of *A*. *sinensis* to enrich the genomic resource for further conservation and evolution researches.

The fresh leaves were collected from a ca. 30-year-old *A*. *sinensis* tree growing in a private botanical garden in Chia-Yi, Taiwan. The voucher specimens (HAST#142367) were deposited in the Herbarium of Academia Sinica, Taipei, Taiwan. Genomic DNA was extracted from fresh leaves by using a CTAB-based protocol (Stewart and Via 1993). A paired-end library was constructed and then sequenced using the Illumina HiSeq 4000 sequencer (Illumina, San Diego, CA). The 49.36 million clean paired-end reads were assembled and analyzed through the use of CLC Genomics Workbench 12.0 (www.qiagenbioinformatics.com/), Bowtie2 (Langmead and Salzberg 2012), and SAMtools 1.9 (Li et al. 2009). The entire cp genome of *A*. *sinensis* has been annotated by DOGMA (Wyman et al. 2004), tRNAscan-SE 1.21 (Schattner et al. 2005) and BLAST searches (http://blast.ncbi.nlm.nih.gov/Blast.cgi). The cp genome sequence of *A*. *sinensis* was submitted to DDBJ with the accession number of LC491571.

The complete cp genome of *A*. *sinensis* has a circular structure with 174,852 bp in length that is similar to those of *Aquilaria yunnanensis* (174,885 bp; NCBI accession number: NC_036940), *Aquilaria crassna* (174,830 bp; NCBI accession number: MK779998), and *Aquilaria malaccensis* (174,832 bp; NCBI accession number: MH286934). The *A*. *sinensis* cp genome contained two inverted repeat regions of 42,103 bp each, separated by a large single-copy region of 87,331 bp and a small single-copy region of 3,348 bp. The contents of A, T, C, and G in the *A*. *sinensis* cp genome were found to be 31.5, 36.7, 18.7, and 18.0%, respectively. The *A*. *sinensis* cp genome encoded 8 rRNAs, 39 tRNAs, and 90 proteins. Of those genes, *rps8* and *rps19* were proposed to have a putative start codon, GTG.

**Figure 1.**
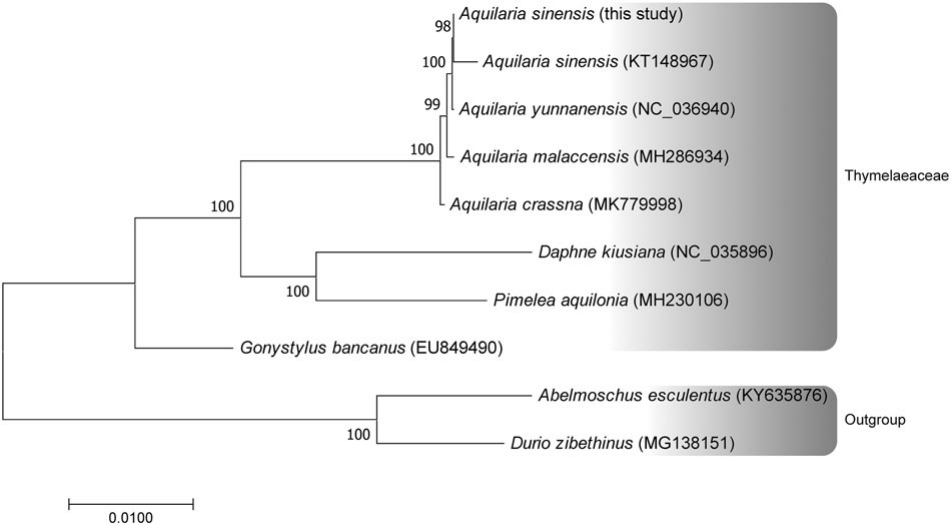
The cp genome phylogenetics of Thymelaeaceae. A maximum-likelihood tree inferred from analysis of a data set containing 43 concatenated protein-coding genes in 10 plastomic taxa by use of the GTR + I + G model. Numbers at each node indicate bootstrap support. GenBank accession numbers of the species used in this phylogenetic tree are enclosed in brackets.

The DNA sequences of 43 common protein-coding genes from eight Thymelaeaceae were concatenated for phylogenetic analysis. Two other Malvales, *Abelmoschus esculentus* and *Durio zibethinus*, were selected as the outgroup. The best-fit, GTR + G + I, nucleotide substitution model was employed in plant cp genome phylogenetic reconstruction. A maximum-likelihood tree was reconstructed using MEGA7 program (Kumar et al. 2016) with 1000 bootstrap replicates. The five examined species of *Aquilaria* appeared a monophyletic group with robust support (Figure 1). Our study would provide useful genomic resource for further studies in *A*. *sinensis* conservation and evolution.
